# The Clinic

**Published:** 1897-10

**Authors:** E. P. Beadles

**Affiliations:** Danville, Va.


AMERICAN JOURNAL

—OF—

DENTAL SCIENCE.

VOL. XXXI. BALTIMORE, OCTOBER, 1897. No. 6.

ARTICLE I.

### THE CLINIC.

BY E. P. BEADLES, D. D. S., DANVILLE, VA.

Ocular demonstration is the best teacher. When a child sees a thing he will remember it easier than if only told about it. Men are simply grown-up children. A desire to learn something new is the foundation upon which all our dental associations rest. The clinic has been the favorite method of imparting knowledge. Many men attend the annual meeting for the clinic alone. They can read the papers and discussions in the journals; but cannot see the operations. This being true, the very best methods for conducting clinics should be devised. It is evident that the plans which have been in use by most of our societies have served their day. With a small number present, each man may be able to see what is going on; with a large number present nobody gets any satisfaction, the operator is crowded and worried, often failing to do himself justice.

The idea should be, not to have so many clinics, not so many operators; but good ones. Some men can teach, others cannot. There is a secret in being able to impart knowledge, to explain a thing so that the hearer may go home and perform the thing for himself. If those who have our clinics in charge for this year will select a few good men of known experience as operators and with ability to tell what they know, and will carry out the following rules, success will be theirs.

1. Have provided only one chair, and have that placed upon a raised platform near the president's seat.

2. Have only one clinic in progress at one time.

3. Let the society be in regular session during the clinic, with the president and other officers in their places.

4. Let each operator furnish beforehand to the manager of the clinic a list of whatever he needs to carry out his work.

5. Have placed on the platform a good blackboard.

Now with the members in their places the clinician is introduced and his intended operation announced. With the patient in the chair and with free use of the blackboard, he explains fully what he intends to do At a certain time those who wish to examine the case are allowed to come up, one or two at a time; this over, the operator proceeds with his work, If it will consume some time another subject may be taken up by the association, business transacted or a paper read, with the understanding that the operator can have the attention of the members whenever he desires it.

We are not students at college, and do not need to see all operations right through in order to be able to do them; a suggestion, an illustration, an explanation may be all that is needed in many cases, or a question asked and answered when all can hear at once. The manager will be able to do his work with greater satisfaction, having only one clinic on hand at a time. By the old plan he is at the beck and call of every operator, and is wanted in a dozen places at once. But possibly the most important point covered by the adoption of this plan will be the effect upon the operator. As it is now, good men often refuse to clinic, well knowing the difficulties under which they must labor. The operator knows that at best he can only explain his method to a few, and perchance the man next to him may be showing something very novel and has the "crowd." The first operator has spent time and trouble to get ready, and is showing something really valuable, but there is no one to see it. He completes his case in silence, steals off out of sight wishing "he hadn't."

By the plan here outlined no one who has been invited to clinic will be neglected. He will have the ear of the entire association, he will feel stimulated to do his best, and knowing this will prepare his best. Our prominent men will feel it worth their while to accept invitations to clinic, everything will be done orderly and "satisfaction guaranteed."—*Atlanta Dental Journal.*

---

ARTICLE II.

